# Plant Growth Promoting Potentials of Beneficial Endophytic *Escherichia coli* USML2 in Association with Rice Seedlings

**DOI:** 10.21315/tlsr2021.32.1.8

**Published:** 2021-03-31

**Authors:** Munirah Tharek, Dzulaikha Khairuddin, Nazalan Najimudin, Amir Hamzah Ghazali

**Affiliations:** 1 School of Biological Sciences, Universiti Sains Malaysia, 11800 USM Pulau Pinang, Malaysia; 2 Department of Water Resources and Environmental System, Faculty of Civil Engineering, Universiti Teknologi MARA, 40450 Shah Alam, Selangor, Malaysia

**Keywords:** Endophytic Colonisation, Plant Growth Promotion, *Escherichia coli* USML2, PGPE, Kolonisasi Endofitik, Penggalak Pertumbuhan Tanaman, *Escherichia coli* USML2, PGPE

## Abstract

An endophytic *Escherichia coli* USML2 originally isolated from the inner part of an oil palm (*Elaeis guineensis* Jacq.) leaf tissue was inoculated to rice seedlings to investigate its ability in colonising plant inner tissues and promoting growth. Infection of *E. coli* USML2 was initiated by colonisation on the root surface, invasion of the interior root system followed by endophytic spreading. Inoculation of *E. coli* USML2 in the rice rhizosphere zone resulted in a significant increase in leaf numbers (33.3%), chlorophyll content (33.3%), shoot height (34.8%) and plant dry weight (90.4%) of 42 days old rice seedlings as compared to the control. These findings also demonstrated the ability of *E. coli* USML2 to spread endophytically which serves as a beneficial strategy for the bacterium to colonise the host plant and gain protection against adverse soil conditions. The genome of *E. coli* USML2 had also revealed predicted genes essential for endophytic bacterial colonisation and plant growth promotion which further proven potentials of *E. coli* USML2 as Plant Growth Promoting Endophyte (PGPE).

## Abstract (ms)

*Escherichia coli* USML2 endofitik yang asalnya dipencilkan daripada bahagian dalaman tisu daun kelapa sawit (*Elaeis guineensis* Jacq.) telah diinokulat kepada anak benih padi untuk menilai kemampuan *E. coli* USML2 untuk kolonisasi tisu dalaman dan menggalakkan pertumbuhan perumah. Jangkitan *E. coli* USML2 bermula dengan kolonisasi bakteria pada permukaan akar, pencerobohan tisu dalaman akar dan diikuti oleh penyebaran endofitik. Inokulasi *E. coli* USML2 di zon rhizosfera padi mengakibatkan peningkatan jumlah daun yang ketara (33.3%), kandungan klorofil (33.3%), tinggi tunas (34.8%) dan berat kering (90.4%) anak benih padi berusia 42 hari berbanding kawalan. Penemuan ini juga menunjukkan kemampuan *E. coli* USML2 untuk tersebar secara endofitik yang berfungsi sebagai strategi bermanfaat bakteria untuk kolonisasi tanaman dan mendapat perlindungan terhadap keadaan tanah yang tidak sesuai. Maklumat genom *E. coli* USML2 juga telah mengungkapkan ramalan gen yang penting untuk kolonisasi bakteria endofitik dan penggalak pertumbuhan perumah dan mengukuhkan potensi *E. coli* USML2 sebagai Endofit Penggalak Pertumbuhan Tanaman (PGPE).

**Highlights**

- Annotation of *Escherichia coli* USML2 genome sequence revealed genes involved in bacterial motility towards endophytic colonisation and plant growth promotion.
- Demonstrate the ability of *E. coli* USML2 in plant growth promotion and *in planta* spreading of rice seedlings.
- *E. coli* USML2 as a genetically amenable endophyte that can be employed in a sustainable agriculture practice.

## INTRODUCTION

*Escherichia coli* is a harmless natural inhabitant of the gastrointestinal tract of animals and humans (Ingerson-Mahar & Reid 2011). Its dispersal into the environment is due to release of faecal matter from the host. These continuous bulk transfers would result into stable population of *E. coli* in secondary habitats such as the soil (Dublan *et al.* 2014). Therefore *E. coli* was presumed not to be a normal inhabitant of the soil and has been used as an indicator of faecal pollution (Nautiyal *et al*. 2010; Dublan *et al.* 2014). However, discovery of indigenous *E. coli* strains in undisturbed soils of seven uninhabited areas in India with temperatures ranging from −10°C to 45°C demonstrated that *E. coli* is a member of the natural soil biota. Ubiquity of *E. coli* in these diverse soils clearly suggested that it should be treated as native soil borne bacteria (Nautiyal *et al.* 2010). Occurrence of *E. coli* in soils is due to its ability to survive, adapt and actively grow in extra-intestinal environments (Nautiyal *et al.* 2010; Ingerson-Mahar & Reid 2011; Méric *et al.* 2013; Blount 2015). Thus, it was hypothesised that persistence of *E. coli* for long periods of time in tropical, subtropical and temperate environments was in response to available nutrients in soil. Substantial nutrient supplies in soil are also provided from excretion of root exudates that consist of carbohydrates, amino acids, organic acids, vitamins and phytohormones acting as chemoattractants to which bacteria respond (Narula *et al.* 2009; Pedraza *et al.* 2009; Nautiyal *et al.* 2010; Berg *et al.* 2013). Thus, this condition attracts broad diversities of soil bacteria to live in the rhizosphere zone and consequently, *E. coli* strains must compete for survival (Berg *et al*. 2013; Dudeja & Giri 2014). This is probably the reason why *E. coli* has been frequently found endophytically in interior plant tissues of plants where the surrounding environment is free from faecal contamination (Dublan *et al.* 2014). Moreover, interior plant tissues also offer a less competitive niche for their survival and persistence (Berg *et al.* 2013). Méric *et al.* (2013) claimed that presence of *E. coli* in inner parts of leaves is believed to gain relative protection against adverse conditions present on external surfaces of plants. In fact, interior tissues of the host plant have become a favourable habitat for bacteria due to the availability of abundant nutrients and its environmental stability (Ikeda *et al.* 2010). Hardoim *et al.* (2008) claimed that *E. coli* with the ability to live and persist *in planta* are known as endophytes.

Plant Growth Promoting Endophytes (PGPE) usually possess close relationships with their host plant (Berg *et al.* 2013) and are able to promote growth, health and development of the host (Taghavi *et al.* 2010). However, knowledge about any potential beneficial effects of endophytic *E. coli* strains on its host plant is very much limited. In fact, the first and only report on plant growth promoting effects of *E. coli* was demonstrated by Nautiyal *et al.* (2010). It was reported that endophytic colonisation of *E. coli* exhibited significant growth promotion of 60-days-old maize (*Zea mays* cv. Arkil) with increased root length, shoot length and plant dry weight when compared with uninoculated seedlings. Hence, it revealed the potential of *E. coli* as a plant growth promoting endophyte. However, *E. coli* strains used by Nautiyal *et al.* (2010) were soil borne which was introduced anthropically by immersing surface sterilised maize seeds in suspension of *E. coli* cells (8 log10 CFU/mL). Additionally, study by Méric *et al.* (2013) which have isolated *E. coli* strains from interior plant tissues have not reported any information on the effect of its endophytic presence on growth of its host plant. However, earlier findings by Tharek *et al.* (2011) have successfully isolated *E. coli* USML2 from inner leaf tissues of surface sterilised healthy oil palm (*Elaeis guineensis* Jacq.). The isolate was initially identified as an *Enterobacter* species and it was shown to be able to infect and establish an endophytic relationship with rice seedlings *Oryza sativa* (MR220). The strain was selected as the most suitable candidate to investigate further on how an endophytic plant associated *E. coli* could potentially promote plant growth and understand the implicated responsible genes involved. Use of *E. coli* in this study is also an advantage due to its rapid cell division rate and rapid adaptation to the environment. Furthermore *E. coli* also requires simple nutritional prerequisites and has well established genetics and completed genome sequence (Brambilla 2014). Moreover, to our knowledge, no study has been conducted to discover the potential of indigenous plant-originated *E. coli* on plant growth promotion abilities. In addition, this is the first report on endophytic plant associated *E. coli* which aids in understanding the fundamental processes of plantbacterial interaction. Thus, the aims of this study are as follows: (i) to observe the ability of an *E. coli* USML2 in endophytic colonisation and plant growth promotion in association with rice seedlings and (ii) to predict all essential genes of *E. coli* USML2 which are involved in promoting endophytic colonisation and plant growth enhancement.

## MATERIALS AND METHODS

### Non-Pathogenic Plant Associated Endophytic *E. coli* USML2

The strain *E. coli* USML2 used in this study was isolated from the inner segments of leaf tissues of surface sterilised oil palm plant (*Elaeis guineensis* Jacq.). The isolate, which was initially identified as an *Enterobacter* species, was shown to be able to infect and establish an endophytic relationship in rice seedlings *Oryza sativa* variety MR220 (Tharek *et al.* 2011). However, based on more in-depth molecular identification analysis via polymerase chain reaction (PCR) and amplification of full length 16S rRNA gene fragment (~1.4 kb), the isolate was re-identified as a plant-associated *E. coli* USML2 (Tharek *et al.* 2017). The genome sequence data was deposited to NCBI with GenBank accession number of CP011124. The genome exhibited no genes involved in pathogenicity which reveals this strain as a non-pathogenic *E. coli*. Additionally, *E. coli* USML2 is in the same clade as *E. coli* K-12 sbstr. MG1655, *E. coli* K-12 DH10B and *E. coli* BW2952 which are strains known as non-pathogenic to humans. This strain was deposited at the Microbial Culture Collection Unit, Institute of Bioscience, Universiti Putra Malaysia, with the accession number of UPMC432. The whole genome shotgun project has been deposited at DDBJ/EMBL/Genbank under the accession number CP011124 (Tharek *et al*. 2017).

### Predicted Genes for Promoting Endophytic Colonisation and Plant Growth Enhancement of *E. coli* USML2

The genome sequence data of *E. coli* USML2 with GenBank Accession Number CP011124 was used to annotate and predict genes which are related to colonisation and plant growth promotion abilities (Tharek *et al.* 2017). These genes involved in metabolic pathways were generated by Kyoto Encyclopaedia of Genes and Genomes (KEGG) embedded in RAST server (Kanehisa *et al.* 2016; Aziz *et al.* 2008; Overbeek *et al.* 2014).

### Construction of *E. coli* USML2 *flhC*::*Km^r^* Mutant

**Figure 1:**
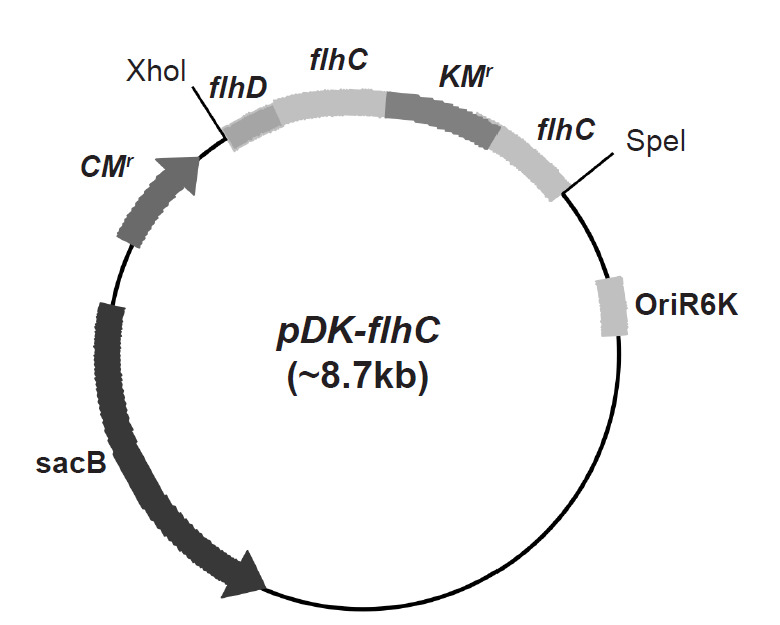
*pDK-flhC*, constructed in this study.

A mutant strain of *E. coli* USML2 has been constructed to verify endophytic colonisation potentials of the isolate. Mutagenesis of a gene involved in flagella biogenesis of *E. coli* USML2 was performed and the *flhC* gene (located within the *flhDC* operon), was selected for mutation. This operon codes a vital positive regulator which controls the initiation of flagellum biosynthesis. An *flhC*::*Km^r^* mutant was constructed in a suicide vector (pDM4) (Yao *et al.* 2007). Initially, *flhDC* genes were amplified from the chromosomal DNA of *E. coli* USML2 and ligated into pLUG-Prime^®^ plasmid vector resulting in pPM10-*flhC*. Insertion of kanamycin resistant gene (*Km^r^*) amplified from pKEM100 (Hasan *et al.* 2004) within pPM10-*flhC* resulted into construction of pPM20-*flhC.* Amplified *flhC::Km^r^* fragment was digested and cloned into pDM4 prior to transformation into *E. coli* S17-λpir. Transformants were selected on Broomfield agar supplemented with kanamycin and chloramphenicol (Blomfield *et al.* 1991). The resulting suicidal plasmid *pDK-flhC* (Fig. 1) was transformed into *E. coli* USML2 and plated on Broomfield agar supplemented with the kanamycin. Selected purified colonies were then grown overnight in broth containing kanamycin to allow plasmid-chromosome recombination to occur at the homologous site. Following this, selected colonies were tested for sucrose sensitivity to screen for mutant strains. PCR primers designed from DNA sequences located outside the *flhDC* gene were then used. Purified PCR products were sequenced by MyTACG Bioscience Enterprise, Inc. (Malaysia) according to the Sanger sequencing procedures. The mutant strain of *E. coli* USML2 *flhC::Km^r^* was non-motile in contrast with the wild type strain and no flagella formation was observed.

### Inoculum Preparation

Wild type strain of *E. coli* USML2 as well as a newly constructed *E. coli* USML2 *flhC::Km^r^* mutant (impaired flagella biogenesis) and *Azospirillum brasilense* Sp7 (ATCC 29145) (a plant growth promoting rhizosphere bacterium) (Tarrand *et al.* 1978) were used in this investigation. All bacterial strains tested were grown in 100 mL nutrient broth (NB)(Oxoid^TM^) in a 250 mL Erlenmeyer flask and incubated at 37°C on an orbital shaker (180 rpm) for 24 h. The bacterial strains were collected by centrifugation (4 500 xg, 15 min) and washed twice with normal saline (0.15M NaCl) for inoculum preparation. A concentration of the inoculum was then adjusted to 10^8^ CFU/mL with normal saline, based on optical density at 600 nm and was confirmed by plate counting as described by Compant *et al*. (2005).

### Verification of Successful Endophytic Colonisation of *E. coli* USML2 in Tissues of Rice Seedlings Grown Under Gnotobiotic Conditions

Gnotobiotic 7-day-old rice seedlings were transferred aseptically into sterilised glass tubes (20 cm in height, 2.5 cm in diameter) containing 2 mL of half-strength N-free Yoshida medium (Yoshida *et al.* 1976). A total of 1 mL bacterial (10^8^ CFU/mL) culture was inoculated directly onto the roots using sterilised pipette tip (10 mL). Both inoculated (+ *E. coli* USML2 or + *E. coli* USML2 *flhC::Km^r^* mutant) and uninoculated rice seedlings were incubated under 12 h photoperiods for 24 h.

Successful rhizoplane and endophytic colonisation (internal tissues of roots, stems and leaves) of both wild type and mutant strains of *E. coli* USML2 of rice seedlings were verified via plate counting method (Muthukumarasamy *et al.* 2006) and PCR analysis of two important housekeeping genes (*groEL* and *ftsZ*) and *marB*. Primers for these genes were designed based on sequence available in Scientific Database for the Bacterium *Escherichia coli* K-12 MG1655 (EcoCyc). Prior to PCR amplification of respective genes, DNA extraction was performed using G-spin™ Total DNA Extraction Mini Kit (iNtRON Biotechnology, Inc, Korea) as per manufacturer’s instruction. The PCR amplification was carried out using the following primers: *groEL*_F (5’-ATG GCA GCT AAA GAC GTA AAA TTC GGT AAC-3’), *groEL*_R (5’-TTA CAT CAT GCC GCC CAT GCC ACC-3’), *ftsZ*_F (5’-ATG TTT GAA CCA ATG GAA CTT ACC AAT G-3’), *ftsZ*_R (5’-TTA ATC AGC TTG CTT ACG CAG AAT GC-3’), *marB*_F (5’-ATG AAA CCA CTT TCA TCC ATA-3’) and *marB*_R (5’CTA CAT AGC GTG TTG ATT ATA ATA G-3’). Terra™ PCR Direct Polymerase Mix (Clontech® Laboratories, Inc.) was used to directly amplify the selected genes which were extracted from 2.0 mm slices of root, stem and leaf of treated rice seedlings, according to manufacturer’s recommendation. PCR amplifications were carried out with a Bio-Rad thermocycler (Hercules, California, USA) under the following conditions: an initial denaturation at 98°C for 2 min, 30 cycles of denaturation at 98°C for 10 s each, annealing at 55°C for 15 s and 30 cycles, extension at 68°C for 1 min, 30 cycles and final extension at 68°C for 5 min. The purified DNA was then sent for sequencing to a service provider, MyTACG Bioscience Enterprise (Selangor, Malaysia). The sequences obtained were compared to those in the GenBank database using the National Center for Biotechnology Information (NCBI) website (http://blast.ncbi.nlm.nih.gov/Blast.cgi) via nucleotide-nucleotide Basic Local Alignment Search Tool (BLASTn) program (Altschul *et al.* 1990).

Visual confirmation of rhizoplane and endophytic colonisation of inoculum tested on plant samples were also viewed under scanning electron microscope (SEM) and transmission electron microscope (TEM) after 24 h of inoculation. For SEM observations, a total of 1 cm length of the main root was excised with a sterile razor blade. Samples were mounted on stubs for SEM, coated with aurum (SEM Coating System, SC515) and viewed under a FESEM Zeiss SUPRA 50VP (Lim *et al.* 2016). Additionally, endophytic colonisation of inoculum tested was also observed under transmission electron microscopy (TEM). Samples of 10 mm segment from respective surface sterilised root, stem and leaf of inoculated seedlings were prepared, fixed, stained according to Fan *et al*. (2011) and viewed under a TEM Philips CM12 (Carl Zeiss AG Oberkochen, Germany).

### Growth of Rice Seedlings Inoculated with *E. coli* USML2 Under Glasshouse Condition

Rice seeds (*Oryza sativa* L. cv MR220) were surface sterilised according to Gyaneshwar *et al.* (2001) prior to germination. Surface-sterilised seeds were transferred onto 1% (w/v) sterile water agar (Microbiological agar Merck, Darmstadt, Germany) and left to germinate for 3 days in the dark at room temperature followed by 12 h photoperiod incubation until day 7. The gnotobiotic 7-days-old rice seedlings were transplanted into undrained pots (1 seedling per pot) containing 0.5 kg of sterilised river sand supplemented with half-strength N-free Yoshida medium (Yoshida *et al.* 1976). The seedlings were maintained in the plant house of School of Biological Sciences, USM under ambient conditions. Initially, the solution level (half-strength N-free Yoshida medium) was adjusted to slightly above the sand level. Bacterial suspensions of inoculation treatments were individually pipetted onto the sand surface area closed to the seedlings. At 3 days after treatment (DAT), the level of half-strength Yoshida medium was increased to 3 cm above the sand level and maintained throughout the experiment. The treatments applied for this experiment were: (1) + inorganic N (NH_4_NO_3_) (40mg/L, dissolved in distilled water), (2) without inorganic N (distilled water only), (3) + *E. coli* USML2 (overnight grown culture in nutrient broth (NB), 5 mL/pot at 10^8^ cfu/mL), (4) + *E. coli* USML2 *flhC::Km^r^* as control inoculum (overnight grown culture in nutrient broth (NB), 5 mL/pot at 10^8^cfu/mL), and (5) + *A. brasilense* Sp7 (overnight grown culture in nutrient broth (NB), 5 mL/pot at 10^8^ cfu/mL).

Non-destructive assessment of plant growth parameters (shoot length, leaf numbers and chlorophyll content) were observed weekly until 42 DAT. The leaf greenness (SPAD values) of youngest fully expanded leaves for each seedling was recorded using a portable leaf-chlorophyll meter (MINOLTA^TM^ SPAD-502) (Neufeld *et al*. 2006). The actual leaf chlorophyll content was determined based on the standard curve of SPAD values and total leaf chlorophyll content of the seedlings (mg chlorophyll mg^−1^ leaf fresh weight) (Amir *et al.* 2001). In addition, at day harvest (42 DAT), destructive growth assessment of the seedlings which include dry weight of shoot and root was executed.

## STATISTICAL ANALYSIS

The experiments were laid out in a completely randomised design (CRD) with five replications for each treatment combination. Data for microbial colonisation and plant growth observations were statistically analysed by using SAS software (SAS Institute, North Carolina, USA). Following the analysis of variance procedure, differences among treatment means were determined according to Tukey-Kramer’s Honestly Significant Difference (HSD) test, *P* < 0.05.

## RESULTS

### Endophytic Colonisation of *E. coli* USML2 in Association with Rice Seedlings

*E. coli* USML2 was able to colonise the rhizoplane and internal tissues of root, stem and leaf (endophytically) of the host plants grown under aseptic conditions (Table 1). Viable cells of *E. coli* USML2 increased from 12 h to 24 h after inoculation. At 12 h of incubation, viable cell count was higher in the internal root tissues (21.0 × 10^4^ CFU/g^−1^ (wet wt)), followed by stem (0.6 × 10^4^ CFU/g^−1^ (wet wt)) and leaf (0.02 × 10^4^ CFU/g^−1^ (wet wt)). Similar results were observed at 24 h after inoculation.

However, for rice seedlings inoculated with mutant strain *E. coli* USML2 *flhC::Km^r^*, the colonisation was observed only on the rhizoplane and internal tissues of roots. Even though colonisation of the plant parts increased from 12 h to 24 h after inoculation, the viable cells were lower as compared to those in rice seedlings inoculated with wild type strain. At 12 h after inoculation, mutant strain exhibited 44.4% and 56.2% lower viable cell count on the rhizoplane and internal root tissues, respectively. Similar trends were observed at 24 h after inoculation which showed 79.6% and 45.3% lower viable cell count in rhizoplane and internal root tissues compared to the wild type strain. However, mutants failed to colonise the internal tissues of aerial plant parts (stem and leaves). Presence of wild type and mutant strains of *E. coli* USML2 was further verified via PCR amplification of *groEL, ftsZ* and *marB* genes in *E. coli*. Results for the verification study matched results from the viable cell count where it showed that wild type strain successfully colonised all plant parts tested. While, the mutant strain only colonised the rhizoplane and internal tissues of roots within 24 h. No gene was detected in the internal tissues of aerial plant parts showing that mutant strain failed to colonise the internal tissues (endopytically) of both stem and leaves.

**Figure 2:**
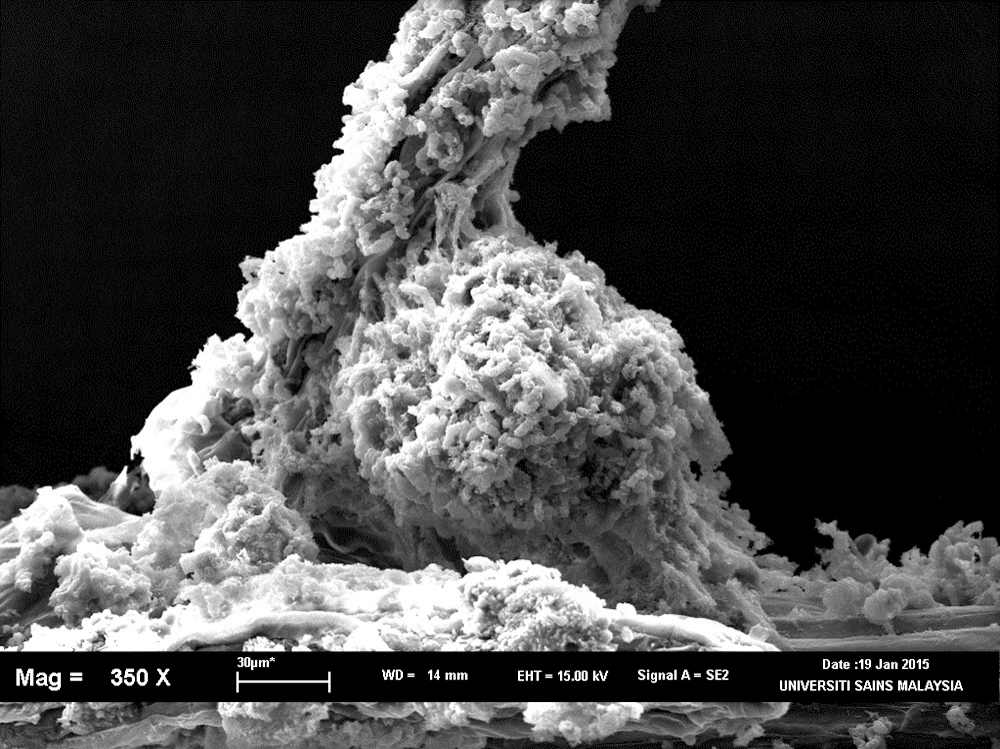

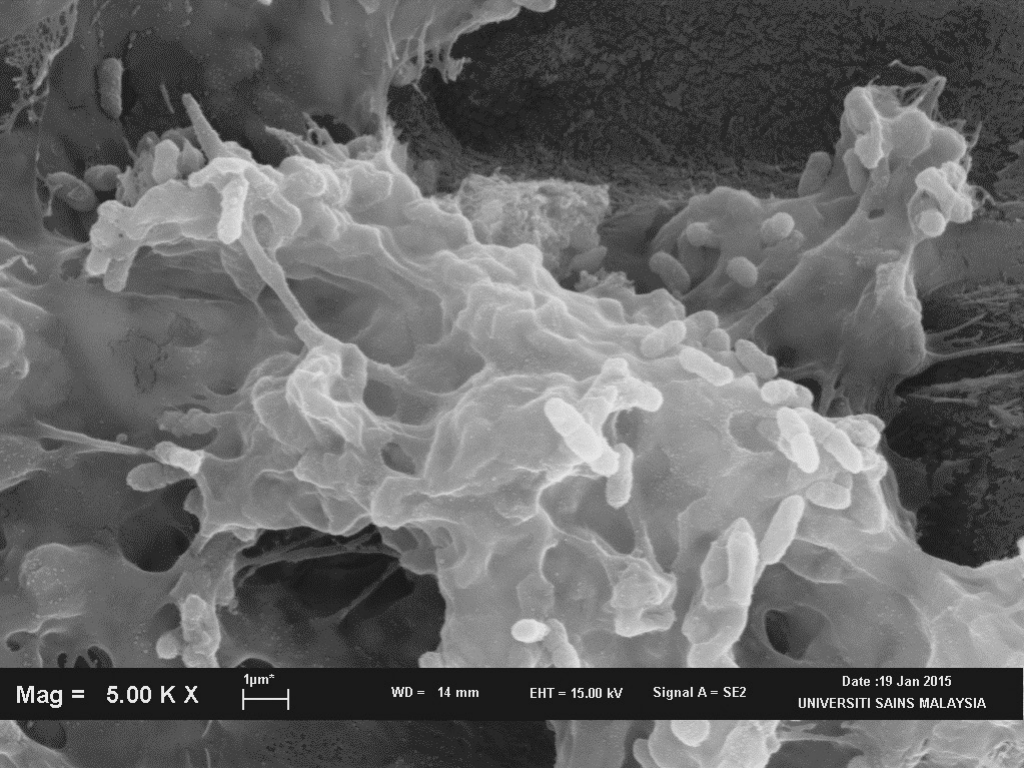

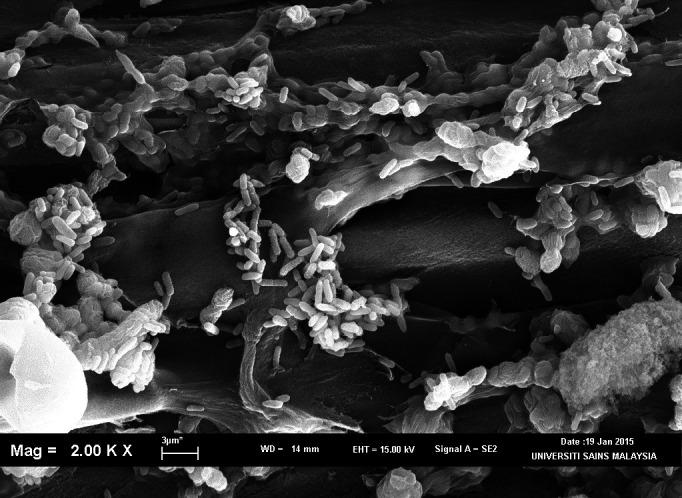
(A) Inoculated roots was covered abundantly by bacteria microcolonies at the lateral root junction and root hairs; (B) Roots of rice seedlings showing presence of microcolonies within layers of exopolysaccharide; (C) SEM micrographs of rhizoplane of rice seedlings inoculated with *E. coli* USML2 *flhC::Km^r^* mutant.

Microscopic analysis under SEM showed that the rhizoplane was colonised by rod shaped bacteria in the form of microcolonies (Figs. 2A and 2B). Colonisation appeared to be more abundant at the base of the lateral root junctions and on root hairs (Fig. 2A). Layers of exopolysaccharide were also observed on the rhizoplane (Fig. 2B). The SEM images also revealed presence of mutant strains on the rhizoplane zone (Fig. 2C). Based on the micrographs, presence of bacteria was not uniform on the entire root surface. Colonisation appeared to be more at the root cracks present on the root surfaces.

**Figure 3:**
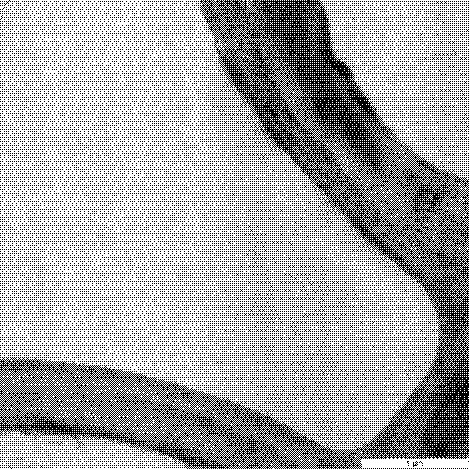

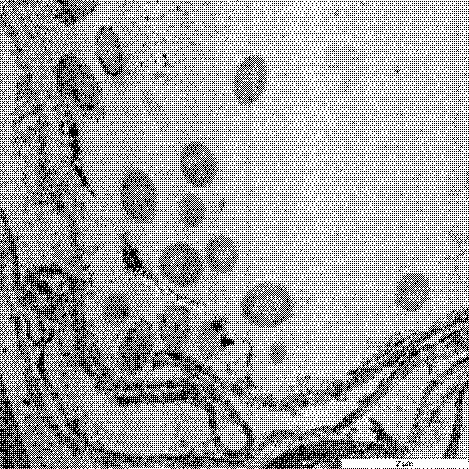

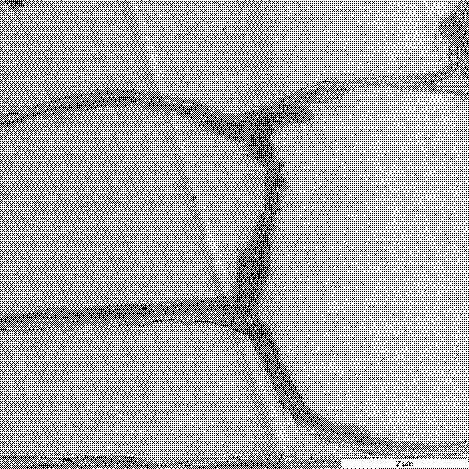

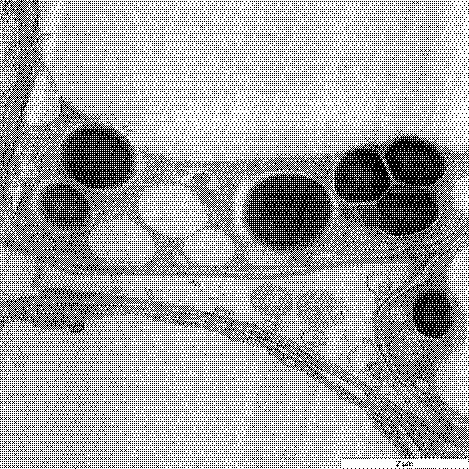

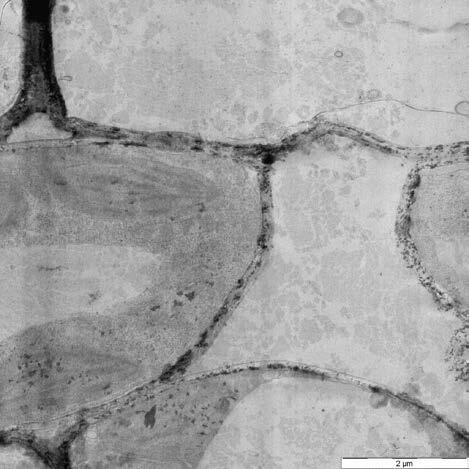

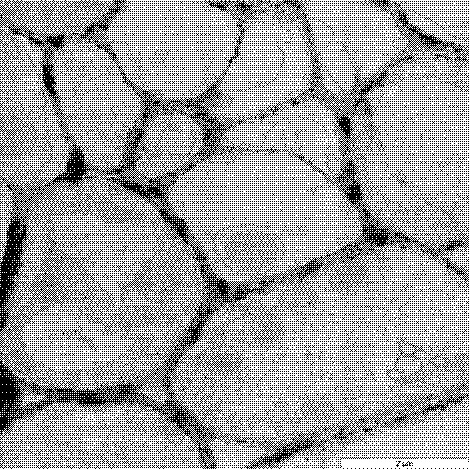

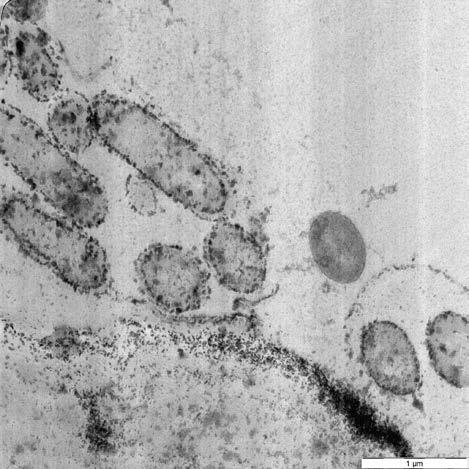
(A) Uninoculated root tissues of rice seedlings exhibits absence of bacterial cells; (B) Inoculated root shows bacterial colonisation in intracellular and intercellular spaces; (C) Uninoculated stem tissues of rice seedlings exhibits absence of bacterial cells; (D) Localisation of *E. coli* USML2 in intracellular and intercellular spaces of stem tissues; (E) Uninoculated leaf tissues of rice seedlings exhibits absence of bacterial cells; (F) *E. coli* USML2 observed in the intracellular spaces of leaf tissues; (G) Cells of *E. coli* USML2 *flhDC::Km^r^* in degraded internal root tissues.

Further analysis under TEM clearly showed presence of bacterial cells in the internal tissues of roots, stems and leaves of the host plants as early as 12 h of inoculation (Fig. 3). The bacterial cells were also observed in all plant tissues after 24 h of inoculation. In the root tissues, localisation of *E. coli* USML2 was observed mainly within intracellular and intercellular spaces of the parenchyma cells. At this stage, the parenchyma cell walls of the inoculated roots were seemed disrupted or degraded (Fig. 3B) in contrast to the intact parenchyma cell wall of the uninoculated plant (Fig. 3A). Bacterial cells were observed in both intercellular and intracellular spaces of the stem tissues (Fig. 3D). In the leaf tissues, *E. coli* USML2 cells were localised only in the intracellular spaces (Fig. 3F). Besides that, proteins were observed in intracellular spaces of the stem tissues (Fig. 3D). These proteins are probably plant cell wall degrading enzymes (PCWDEs) secreted by *E. coli* USML2. Additionally, TEM images also showed the presence of bacteria in internal root tissues inoculated with mutant strains. Besides that, structure of the cell wall was degraded probably due to PCWDEs produced by bacteria present *in planta* (Fig. 3G). As expected, no bacteria were observed using TEM of internal tissues of stem and leaf. This finding supported the results from the bacterial enumeration study (Table 2) where *E. coli* USML2 *flhC::Km^r^* mutant failed to colonise the internal tissues of stems and leaves of its host plant. This finding proves the importance of flagella for microbial motality and initiation of endophytic colonisation.

### Plant Growth Promotion of Endophytic *E. coli* USML2 in Association with Rice Seedlings

**Figure 4:**
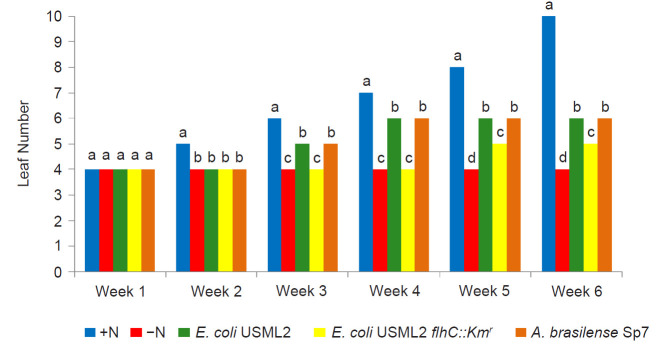

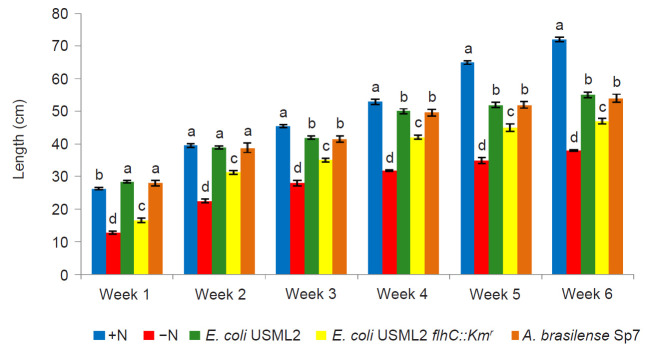

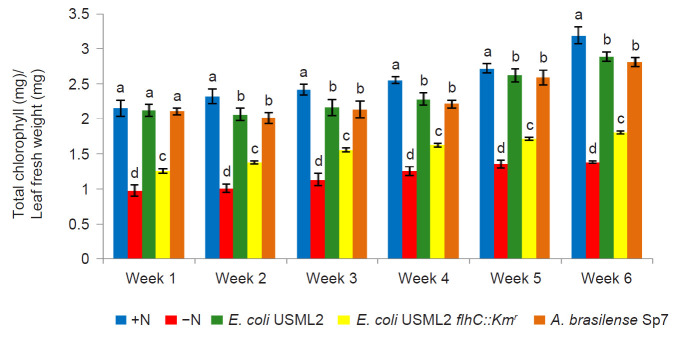

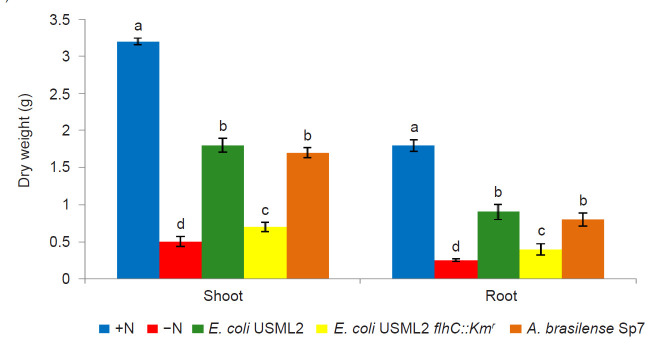
Influence of rhizosphere inoculation on (A) leaf number, (B) shoot length, (C) total chlorophyll and (D) dry weight of 42 days old rice seedlings treated with +N, −N, +*E.coli* USML2, +*E.coli* USML2 *flhC::km^r^* and +*A. brasilense* Sp7. Mean values with the same letters in each week, shoot and root are not statistically different (Tukey HSD, *P* < 0.05).

The results showed that seedlings inoculated with *E. coli* USML2 and *A. brasilense* Sp7 showed equal good growth response (higher leaf number, shoot length and chlorophyll content) compared to the control (-N) (Fig. 4). Interestingly, seedlings inoculated with *E. coli* USML2 and *A. brasilense* Sp7 also showed significantly higher root and shoot dry weight as compared to control treatment (-N) at harvest (D_42_). The results clearly showed that *E. coli* USML2 exhibited similar plant growth promoting effects as established by *A. brasilense* Sp7, a known diazotrophic plant growth enhancer. The seedlings inoculated with the mutant strain (*E. coli* USML2 *flhC::Km^r^*) exhibited significantly poor growth compared to the wild type after 42 DAT (Fig. 4). Those inoculated with mutant strain exhibited significantly lower leaf numbers (16.7%), chlorophyll content (37.4%), shoot height (14.5%) and plant dry weight (59.3%) compared to the wild type strain of *E. coli* USML2.

### Predicted Genes in Promoting Endophytic Colonization and Plant Growth Enhancement of *E. coli* USML2

Annotation generated by RAST server exhibited presence of subsystems related to motility and possible essential steps involved in endophytic bacterial colonisation. Based on the annotation, *E. coli* USML2 is equipped with genes predicted for its active movement towards the plant roots. This is shown by the presence of flagella biosynthesis genes in genome of *E. coli* USML2 which includes the following operons: *flgLKJIHGFEDCBAMN, fliE, fliRQPONMLKJIHGFE, fliTSDCZY, motAB, flhBAE* and *flhDC* (Table 3). The importance of flagella to promote plant endophytic colonisation was successfully validated via mutant strain *E. coli* USML2 *flhC::Km^r^*, with impaired flagella biogenesis. Additionally, bacterial movement was also due to chemotaxis activity which can be described by the presence of chemotaxis genes encoding CheA, CheW, CheR, CheM, CheY, CheZ and methyl-accepting chemotaxis protein. These *che* genes are found within the flagella biosynthesis gene cluster. Following movement of *E. coli* USML2 towards the root *via* motility driven chemotaxis, efficient adhesion on the root surface also plays an important role in successful endophytic colonisation. Non-specific root adhesion by endophytic bacteria usually occurs in the presence of pili. Several genes involved in biogenesis of type IV pili (*pilABCMNOPQ*) were present in *E. coli* USML2 genome. Besides that, *E. coli* USML2 also contains *csg* operon for biosynthesis of curly fibres which is involved in bacteria adhesion to surfaces. Once the bacterial cells are attached to the root surface, they form microcolonies. Bacterial cells within the microcolonies communicate to each other *via* quorum sensing. In *E. coli* USML2, genes required for quorum sensing are also present (*luxR, luxS* and *sdiA*). Communication within the microcolonies would then trigger production of extracellular polymeric substances (EPS). Genes predicted for encoding proteins involved in the production of EPS were also found in *E. coli* USML2. Among the genes discovered were phosphoglucomutase (*pgm*) and phosphomannomutase (*manB*). Presence of these genes predicted the ability of *E. coli* USML2 in EPS production which is essential for firm bacterial anchoring on root surfaces. In addition, the predicted genes involved in biofilm formation such as biofilm regulator protein (*bssR*), outer membrane secretin (*pgaA*), synthesis of deacetylase (*pgaB*), synthesis of N-glycosyltransferase (*pgaC*) and synthesis of auxiliary protein (*pgaD*) are also present.

Firm attachment on the root surfaces is a precondition towards invasion of the plant host. For this purpose, *E. coli* USML2 must be able to degrade the plant cell wall by production of PCWDEs. Since cellulose and pectin are the major composition of the plant cell wall, therefore, production of related enzymes for degradation of both components is essential. Several predicted genes involved in degradation of cellulose and pectin are present in *E. coli* USML2 genome. These include *kdgK*, *kdgR* and *kdgT* genes, which are involved in degradation of pectin. Besides that, the presence of genes encoding for cellulose synthase enzymes (*yhjO*, *bcsABZC*) is also crucial for breakdown of cellulose which further supports the capability of *E. coli* USML2 in invasion of the internal tissues for *in planta* spreading of its host plant.

As for plant growth promoting potentials, like other non-nitrogen fixing plant growth promoting endophytes (PGPEs), *E. coli* USML2 is equipped with predicted genes which aid in nitrogen metabolism. It contains genes essential for dissimilatory and assimilatory nitrate reduction pathways. Among genes present include *narIJHGXL*, *nirCBD* and *ntrABC*. In addition, predicted genes involved in nitrate/nitrate transporter (*narK*) and sensor (*narQ*) were also found (Table 3). *E. coli* USML2 also has the capability to solubilise insoluble phosphate. Its capability in phosphate solubilisation is made possible by presence of genes encoding for acid and alkaline phosphatase enzymes (Table 3). These enzymes release phosphorus from organic compounds present in the soil which later will increase the availability of phosphorus. Furthermore, gene encoding pyrroloquinoline quinone (PQQ)-dependent glucose dehydrogenase (*gcd*) was also predicted (Table 3). The *gcd* gene is essential in biosynthesis of gluconic acid (GA). In fact, secretion of GA by phosphate solubilising bacteria (PSB) is an important mechanism for mineral phosphate solubilisation in Gram-negative bacteria. This is because release of organic acid such as GA chelates the cation bound to phosphate, thus converts it into soluble forms. Additionally, *E. coli* USML2 genome also carries several genes involved in potassium solubilisation (Table 3). These include genes encoding trans-membrane protein (*trkH* and *trkG*), cytoplasmic membrane surface protein (*trkA*), and Kup system potassium uptake protein (*trkD*). Beside these, the genes for the two components transcriptional regulatory proteins of the potassium dihydrogen phosphate (KDP) operon (*kdpE* and *kdpD*) are also present.

Plant growth and morphogenesis are also affected by small molecules or volatile compounds released by microorganisms. These molecules act as phytohormones to activate plant immunity or regulate growth. The strain *E. coli* USML2 also harbours genes involved in the production of auxin and acetoin. The most common naturally occurring plant hormone of the auxin class is indole acetic acid (IAA). The genes for enzyme tryptophanase (*tnaA*) and indole-3-glycerol phosphate synthase (*trpC* and *trpF*) are also present in the *E. coli* USML2 genome. The former is involved in reduction of tryptophan to indole, pyruvate and ammonia while the latter is involved in tryptophan-independent IAA biosynthesis pathway (Edwards & Yudkin 1984; Gaimster & Summers 2015; Panichkin *et al.* 2016). In addition to the genes related to IAA production, *E. coli* USML2 also has the capability to produce acetoin. This is indicated by presence of gene encoding pyruvate dehydrogenase (*aceE*) which converts a small fraction of pyruvate into acetoin.

Ability of *E. coli* USML2 in plant growth promotion is further supported by presence of *acdS* gene encoding for 1-aminocyclopropane-1-carboxylate (ACC) deaminase. This enzyme degrades ACC to form ammonium and α-ketobutyrate which respectively serve as a source of nitrogen and carbon for the host plant. Earlier findings by Yunlei *et al.* (2015), observed essential roles of ACC deaminase (*acdS* gene) in enhancing bacterial heavy metal tolerance of bacteria and plant growth promotion ability. Additionally, S-adenosylmethionine (SAM), which is converted into ACC by the enzyme ACC synthase, is also present. Other than ACC, several genes required for biosynthesis of enterobactin which is a high affinity siderophore were found. The genes *entBCDEFG* which are required for the biosynthesis of enterobactin were found as an operon. Enterobactin is a high affinity siderophore and the integral inner membrane protein EntS is required to export after enterobactin synthesis occurred in the cytoplasm. The iron-enterobactin complex is recovered *via* the ferric siderophore uptake system (ExbDB) and when the complex enters the cytoplasm, iron is extracted by a mechanism requiring the product of the iron ferric enterobactin esterase (*fes*) gene. In addition, *E. coli* USML2 genome also encompasses genes for ferrous iron uptake systems, ferric hydroxamate ABC transporters and outer membrane ferric-related siderophore receptors (TonB-dependent). Genes required for ornithine biosynthesis which is involved in increasing the rate of siderophore production rate are also present. Genes for sulphur metabolism which includes genes for assimilation of inorganic sulphate such as sulphate and thiosulfate binding protein (*cysP*), sulphate transport system permease protein (*cysT* and *cysW*), sulphate or thiosulfate import ATP-binding protein (*cysA*) and several other cys genes (*cysC, cysD, cysH, cysI, cysJ, cysG*) were also predicted.

## DISCUSSION

The results of this research showed that endophytic *E. coli* USML2 has unique characteristics as a PGPE that is able to promote growth of the host plant through associative interactions. This is evidenced by the discovery of predicted PGP genes of *E. coli* USML2 in relation to traits of nitrogen metabolism, phosphate solubilisation, potassium solubilisation, auxin (Indole) production, acetoin production, ACCD production, siderophore production, ornithine biosynthesis and sulphur metabolism. In addition, predicted genes related to promotion of endophytic bacterial colonisation traits such as flagella biogenesis, chemotaxis type IV, pili biogenesis, curly fibres biogenesis, quorum sensing, EPS production, biofilm formation and degradation of cellulose and pectin (PCWDE) has also been found. Results also successfully highlighted endophytic *E. coli* USML2 colonisation of the internal tissues of root, stem and leaf, together with rhizoplane colonisation of the host plants. The inoulation treatment had subsequently promoted plant growth. However, for seedlings inoculated with mutant strain of *E. coli* USML2 *flhC::Km^r^*, no endophytic colonisation in the aerial tissues together with poor growth of rice seedlings were recorded.

The results clearly showed that the initial step of plant colonisation involves bacterial motility. Earlier findings by Turnbull *et al.* (2001) stated that motility is the initial phase of bacterial colonisation which finally contributed towards bacterial survival and attachment in soil particles and roots of the host plant. In this study, a mutant (*E. coli* USML2 *flhC::Km^r^* ) deficient in flagella biogenesis established the importance of flagella in endophytic colonisation of endophytes within the host plant tissues. Similarly, Hockett *et al*. (2013) showed that *Pseudomonas syringae* utilised flagellum-mediated motility to reside the phyllosphere and invade the leaf internals to survive as a pathogen. Genes involved in the biogenesis of flagella and its function consist of a highly complex regulon of at least 50 genes in 17 operons has also been widely investigated (León & Espín 2008; Lee *et al.* 2011; Fan & Macnab 1996). Plants treated with flagella mutant strain had significantly lower growth compared to the wild type strain indicating the importance of bacterial motility towards growth enhancement abilities. The inability of the mutant strain to colonise endophytically in the internal tissues of the aerial plant parts of rice, had affected its effectiveness as a growth promoter. Induced expression of the chemotaxis genes enabled the bacteria to swim to the secreted root exudates with the aid of flagella (Hardoim *et al.* 2008), resulted in bacterial attachment to the root surface followed by formation of microcolonies (Taghavi *et al.* 2010). In this study, efficient rhizoplane colonisation occurred on the root surface especially at the base of the lateral root junctions after 24 h of inoculation. This is probably because root exudates secreted from wounds at root junctions would provide nutrient source for the colonising bacteria and thus create a favourable condition for colonisation. Complex interactions between the bacterial cells and the host plant also resulted in the production of extracellular polymeric substances (EPS) by the microcolonies (Taghavi *et al.* 2010). This process of biofilm formation would also increase bacterial survivability by providing shelter to environmental stress as well as protection from adverse substances such as antibiotics and biocides (Annous *et al.* 2009). The ability of the colonies to produce EPS was also evident before the endophytic bacteria can finally enter the plant (Taghavi *et al.* 2010). Following successful colonisation on the rhizoplane and entry into the root internal tissues, *E. coli* USML2 had spread towards the inner parts of stem and leaves and finally promoted growth of the host plants. This serves as a beneficial strategy for the bacterium to gain protection against adverse conditions present outside the plant (Méric *et al.* 2013) and any potential competition against indigenous microorganism.

Following establishment of endophytic colonisation within the rice seedlings, locally isolated *E. coli* USML2 had successfully promoted growth and development of the host plants probably due to the expression of the PGP genes. Inoculation of *E. coli* USML2 and *A. brasilense* Sp7 showed equal good growth response of the host plants compared to the mutant *E. coli* USML2 *flhC::Km^r^* and control (-N). Wild type *E. coli* USML2 has plant growth promoting traits such as phytohormone and ACC deaminase production. Besides that, gene for siderophore, an iron-chelating complex that makes the metal available to the plant is also present. In addition, *E.coli* USML2 is also capable in solubilisation of phosphate and potassium. These traits stimulate plant growth either by synthesising phytohormones or by promoting nutrients availability (Liu *et al*. 1992; Glick 1995; Bowen & Rovira 1999; Ashrafuzzaman *et al.* 2009). Discovery of numerous predicted PGP genes in this study nominates *E. coli* USML2 as a potential endophytic plant growth promoting candidate. The mutant without flagella failed to spread into the phyllosphere, thus reduced its PGP ability. Similar results were shown earlier by Nautiyal *et al.* (2010) where inoculation of maize seeds with a soil originated *E. coli* NBRIAR3 under microplot conditions had significantly enhanced plant growth when compared to uninoculated control. However, no information on PGP genes of the isolate was highlighted in the findings.

Other findings have also successfully recorded an increase in the number of isolated PGPEs, which has the potential to enhance growth of the host plants. Such PGPEs include species of bacteria from the genera of *Pseudomonas*, *Azospirillum, Azotobacter, Klebsiella, Enterobacter, Alcaligenes, Arthrobacter, Burkholderia, Bacillus* and *Serratia* (Kloepper *et al.* 1989; Okon & Labandera-Gonzalez 1994; Glick 1995; Gururani *et al.* 2012). In fact, PGPE such as *Pseudomonas*, *Bacillus*, *Klebsiella*, *Azotobacter*, *Azospirillum* and *Azomonas* have been widely used as bio-inoculants to promote plant growth and development of agriculture crops (Ahemad & Kibret 2014). Application of PGPEs as inoculants for improving plant growth and yield offers an attractive way to reduce and replace chemical fertilizers, pesticides, and supplements (Stefan *et al.* 2008; Ashrafuzzaman *et al.* 2009). This extends to decrease environmental pollution and health risks originating from excessive use of the agrochemical nitrogen (N) fertilizers to achieve enhanced plant growth (Tambalo *et al.* 2015).

## CONCLUSION

Annotation of *E. coli* USML2 genome sequence revealed presence of genes involved in bacterial motility towards the root, root adhesion, endophytic colonisation, and plant growth promotion. The ability of *E. coli* USML2 in plant growth promotion of rice seedlings and *in planta* spreading was demonstrated. The study also revealed potential of *E. coli* USML2 as a genetically amenable endophyte that can be employed in a sustainable agriculture practice. A further comprehensive transcriptomic and proteomic studies would be able to shed valuable insights towards understanding the complex regulatory pathways involved in plant growth promotion.

## Figures and Tables

**Table 1:** Enumeration of colonised *E. coli* USML2 on the rhizoplane and internal tissues of different plant parts of rice seedlings.

Treatment	Plant part	Viable cell count (10^4^CFU g^−1^ (wet weight))			*E. coli* USML2 Verification of USML2 in planta via PCR								

					*groEL*			*ftsZ*			*marB*		
			
		0h	12h	24h	0h	12h	24h	0h	12h	24h	0h	12h	24h
+ *E. coli* USML2	Rhizoplane	0c	5.4b	843.3a	−	+	+	−	+	+	−	+	+
	Internal root tissues	0c	21.0b	138.1a	−	+	+	−	+	+	−	+	+
	Internal stem tissues	0c	0.6b	19.8a	−	+	+	−	+	+	−	+	+
	Internal leaf tissues	0c	0.02b	0.5a	−	+	+	−	+	+	−	+	+
Uninoculated (Control)	All Plant Parts	0a	0a	0a	−	−	−	−	−	−	−	−	−

*Note*: No bacteria cells were discovered in all plant parts of uninoculated rice seedlings (Control). Mean values of viable cell count with the same letters for each plant part is not statistically different (Tukey HSD, *P* < 0.05).

**Table 2:** Enumeration of colonized *E. coli* USML2 *flhC::Km^r^* on the rhizoplane and internal tissues of different plant part of rice seedlings

Treatment	Plant part	Viable cell count (10^4^CFU g^−1^ (wet weight))			*E. coli* USML2 *flhC::Km^r^* Verification of USML2 in planta *via* PCR								

					*groEL*			*ftsZ*			*marB*		
			
		0h	12h	24h	0h	12h	24h	0h	12h	24h	0h	12h	24h
*+ E. coli* USML2 *flhC::Km^r^*	Rhizoplane	0c	3.0b	172.3a	−	+	+	−	+	+	−	+	+
	Internal root tissues	0c	9.2b	75.5a	−	+	+	−	+	+	−	+	+
	Internal stem tissues	0a	0a	0a	−	−	−	−	−	−	−	−	−
	Internal leaf tissues	0a	0a	0a	−	−	−	−	−	−	−	−	−
Uninoculated (Control)	All plant parts	0a	0a	0a	−	−	−	−	−	−	−	−	−

*Note*: No bacteria cells were discovered in all plant parts of uninoculated rice seedlings (Control). Mean values of viable cell count with the same letters for each plant part is not statistically different (Tukey HSD, *P* < 0.05).

**Table 3:** Predicted genes for promoting endophytic colonisation and plant growth enhancement of *E. coli* USML2 in association with rice seedlings.

	Traits	Genes
Colonisation	Flagella biogenesis	Operons: *flgLKJIHGFEDCBAMN, fliE, fliRQPONMLKJIHGF, fliATSDCZY, motAB, flhBAE* and *flhDC*
	Chemotaxis	cheD, cheA, cheW, mcp, cheM, tap, cheR, cheB, cheY, cheZ, trg
	Type IV pili biogenesis	*pilABCMNOPQ*
	Curli fibres biogenesis	csgCABDEFG
	Quorum sensing	*LuxR, luxS, sdiA*
	EPS production	Phosphoglucomutase (*pgm*), phosphomannomutase (*manB*), bssR, pgaA, pgaB, pgaC, pgaD
	Biofilm formation	Regulator protein (*bssR*), outer membrane secretin (*pgaA*), synthesis of deacetylase (*pgaB*), synthesis of N-glycosyltransferase (*pgaC*), synthesis of auxiliary protein (*pgaD*).
	Degradation of cellulose and pectin (PCWDE)	*kdgK*, *kdgR, kdgT, yhjO*, *bcsABZC, ybhC*

Plant growth promotion	Nitrogen metabolism	*narQ, narI, narJ, narH, narG, narK, narX, narL, nirC, nirD, nirB, ntrB, ntrC, ntrA*
	Phosphate solubilisation	Pyrroloquinoline quinone (PQQ)-dependent glucose dehydrogenase (*gcd*)
	Potassium solubilisation	Trans-membrane protein (*trkH* and *trkG*), cytoplasmic membrane surface protein (*trkA*), Kup system potassium uptake protein (*trkD*), potassium dihydrogen phosphate (KDP) operon (*kdpE* and *kdpD*).
	Auxin (Indole) production	Tryptophanase enzyme (*tnaA*), indole-3-glycerol phosphate synthase (*trpC* and *trpF*).
	Acetoin production	Pyruvate dehydrogenase (*aceE*).
	ACCD production	1-aminocyclopropane-1-carboxylate (ACC) deaminase *(acdS)*, S-adenosylmethionine (SAM) (*sped*).
	Siderophore production	*entB, entE, entC, entF, entD, entS, fes, exbD, exbB*
	Ornithine biosynthesis	*argD, argE, argF, potE, spec*
	Sulfur metabolism	Sulfate and thiosulfate binding protein (*cysP*), sulfate transport system permease protein (*cysT* and *cysW*), sulfate or thiosulfate import ATP-binding protein (*cysA*), taurine production (*tauABCD*).
